# Evaluation of *Get Healthy at Work,* a state-wide workplace health promotion program in Australia

**DOI:** 10.1186/s12889-019-6493-y

**Published:** 2019-02-13

**Authors:** Melanie Crane, Erika Bohn-Goldbaum, Beverley Lloyd, Chris Rissel, Adrian Bauman, Devon Indig, Santosh Khanal, Anne Grunseit

**Affiliations:** 1 Prevention Research Collaboration, The Charles Perkins Centre, Sydney School of Public Health, The University of Sydney, Sydney, NSW 2006 Australia; 2 NSW Office of Preventive Health, Liverpool Hospital, Liverpool, NSW 2170 Australia; 3 The Australian Prevention Partnership Centre, Ultimo, NSW 2007 Australia

**Keywords:** Workplace health promotion, Health promotion, Program evaluation, Mixed methods, Non-communicable disease prevention

## Abstract

**Background:**

Workplace health programs (WHPs) may improve adult health but very little evidence exists on multi-level WHPs implemented at-scale and so the relationship between program implementation factors and outcomes of WHPs are poorly understood. This study evaluated Get Healthy at Work (GHaW), a state-wide government-funded WHP in Australia.

**Methods:**

A mixed-method design included a longitudinal quasi-experimental survey of businesses registered with GHaW and a comparison group of businesses surveyed over a 12-month period. Semi-structured interviews and focus groups with key contacts and employees of selected intervention group businesses and the service providers of the program were conducted to assess program adoption and adaptation.

**Results:**

Positive business-level changes in workplace culture were observed over time among GHaW businesses compared with the control group. Multilevel regression modelling revealed perceptions that employees were generally healthy (*p* = 0.045 timeXgroup effect) and that the workplace promoted healthy behaviours (*p* = 0.004 timeXgroup effect) improved significantly while the control group reported no change in work culture perceptions. Changes in perceptions about work productivity were not observed; however only one third of businesses registered for the program had adopted GHaW during the evaluation period. Qualitative results revealed a number of factors contributing to program adoption: which depended on program delivery (e.g., logistics, technology and communication channels), design features of the program, and organisational factors (primarily business size and previous experience of WHPs).

**Conclusions:**

Evaluation of program factors is important to improve program delivery and uptake and to ensure greater scalability. GHaW has the potential to improve workplace health culture, which may lead to better health promoting work environments. These results imply that government can play a central role in enabling prioritisation and incentivising health promotion in the workplace.

**Electronic supplementary material:**

The online version of this article (10.1186/s12889-019-6493-y) contains supplementary material, which is available to authorized users.

## Background

Globally, non-communicable diseases (NCDs), including heart disease, stroke, cancer and diabetes, account for 71% of all deaths [1]. Many NCD risk factors are preventable through health promotion initiatives to reduce tobacco use, unhealthy diets and alcohol use, and to promote physical activity in the population [1]. Workplaces are a good setting for health promotion initiatives because of the potential reach: over 3.4 billion people make up the global labour force [2]. In Australia, 12 million people, or 95% of the working-age population, are employed [3] and, like other OECD countries, spend on average 36 h per week at work [4].

Workplace health programs (WHPs) are coordinated and comprehensive health promotion strategies comprising of policies, environmental supports and activities in the workplace to engage workers in healthy behaviours and facilitate their wellbeing [5]. WHPs differ from workplace health and safety programs in that the latter are injury-focussed while WHPs tend to focus on lifestyle-related NCD prevention [5]. Benefits to employees of WHPs reported include decreased risk of NCDs and improved health behaviours (e.g., physical activity and nutrition) [6–10]; while benefits to businesses have included improved market value [11] and return on investment [7, 12]. Evidence of the effectiveness of WHPs on productivity is mixed; one recent review [13] was inconclusive while a meta-analysis found limited health and productivity benefits [14].

Evaluating the implementation of WHPs is central to understanding the benefits and the factors which facilitate or inhibit their effectiveness and sustainability [15]. Most evaluation research on WHPs has focused on measuring program outcomes, yet comprehensive evaluation should also capture the implementation process [16, 17]. This is because WHPs are often complex, having multiple components, targeting multiple health behaviours, involving multiple levels of influence within an organisation or addressing multiple determinants [18, 19]. The mechanisms for success of such programs depend on context and so evaluations need to examine contextual factors influencing implementation [20]. Complexity is further increased when programs are implemented at-scale (e.g., state or nation-wide) across multiple workplace settings [21]. Process evaluation becomes particularly important in this case, not only because one intervention may be implemented differently at multiple sites [22] but also because multiple levels of implementation introduce additional layers of complexity [23].

The various aspects of the program, the context it is delivered in, and the levels of implementation involved, contribute to an uncertainty about the impacts the program and how they manifest. This uncertainty produces what known in complex systems science (or systems thinking) as *emergence*, and means that the specific outcomes and the way in which they occur may emerge during implementation process, and will be unknown a priori [24]. Various frameworks for evaluating implementation processes in health promotion have been developed [15, 19, 25, 26]. These emphasise a need to investigate the characteristics of, and interaction between, the intervention, the organisation and the implementer. Where there are multiple levels of implementation, actions at each level need to be included in the evaluation as variability in individual level outcomes may reflect contextual processes [27] such as those occurring upstream at the policy level [28, 29]. At the organisational level, Weiner suggests that implementation effectiveness is subject to (i) the adaption-fit between the program and the organisation; and (ii) employee acceptance of the program (both the end-user and those implementing it in the business) [18]. Complex evaluation theory also emphasises the need for multiple evaluation methods, because neither quantitative nor qualitative approaches provide adequate insight into the implementation of complex programs [20, 22, 30].

In practice, WHPs have tended to be limited in scope (e.g., comprise only an environmental support or address only one health behaviour) [31], and implemented in one or a small number of organisations [32]. Evidence of large-scale WHP implementation and evaluation is scarce: those that have been evaluated have concentrated on quantifying one or a limited number of health impacts at the employee level [33–35]. While changes in health at the employee level should be the ultimate goal, there is often very little evaluation of the mechanisms of change in interventions with multiple levels of implementation or which are delivered at-scale. The purpose of this study was therefore to evaluate the state-wide implementation of a complex WHP in Australia and to assess its short-term impacts at the business level. Specifically, the aim of this study was to evaluate the *Get Healthy at Work* (GHaW) program, a government-funded WHP initiative to reduce workers’ risk of chronic disease.

## Methods

### Intervention

**Fig. 1:**
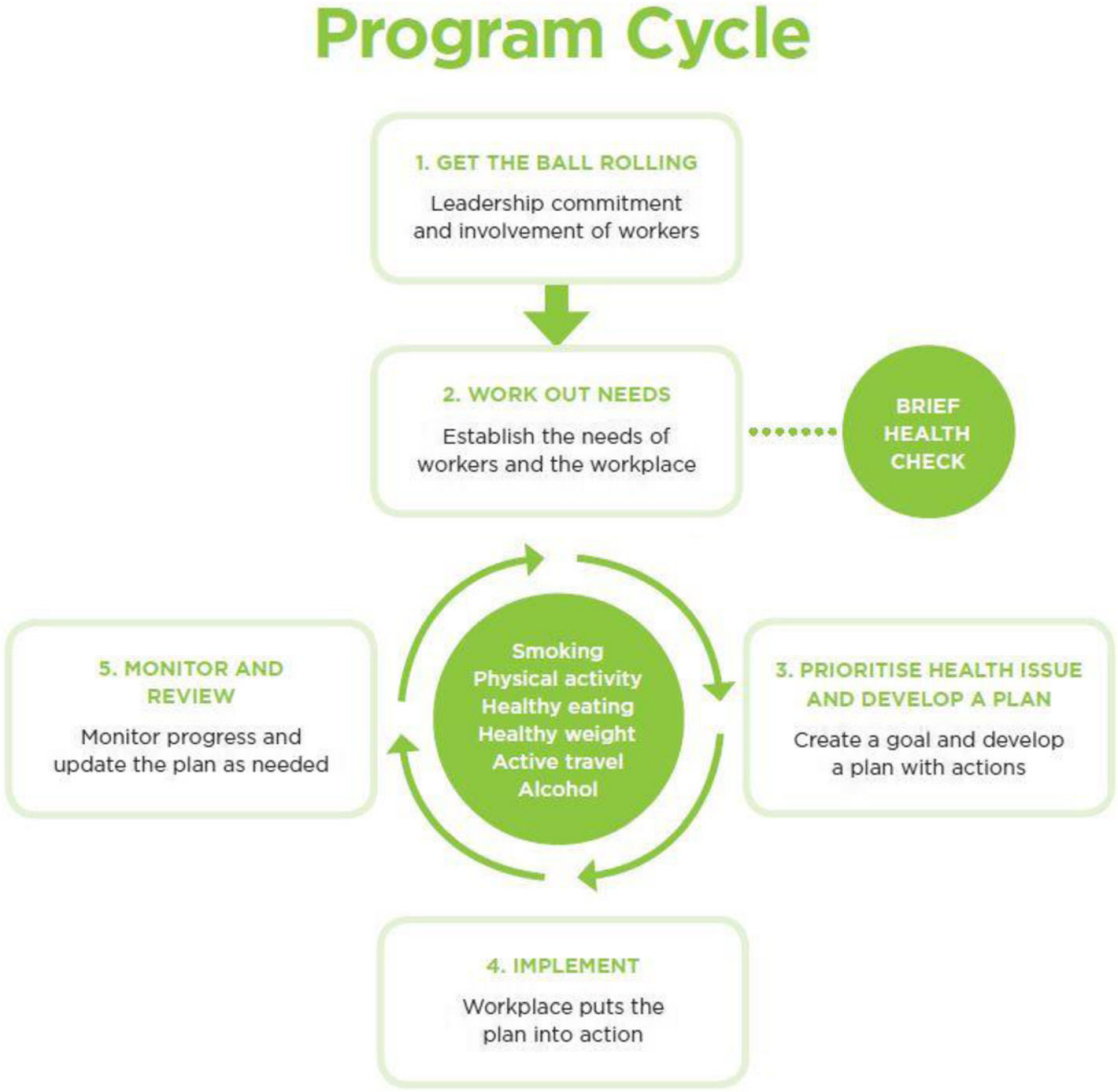
Get Healthy at Work (GHaW) program implementation cycle. Source: Get Healthy at Work: the program cycle (with permission)

GHaW is a state government sponsored comprehensive WHP for workplaces in New South Wales (NSW), Australia [36] developed under the Healthy Workers Initiative [37]. The program consists of health education and action plans designed to enable workplaces to incorporate activities and policies with implementation support, program materials and financial assistance. After registering for the program through an online portal, businesses progress through the GHaW program cycle of assessing employee health risks, implementing action plans and reviewing progress (Fig. 1). They may choose to use the assistance of a service provider or a do-it-yourself (DIY) model online. Once a business has completed an assessment of its health needs and conducted employee brief health checks, the business selects one of six priority health issues (physical activity, healthy eating, smoking, healthy weight, active travel and alcohol) to address as part of their WHP. The business develops and tailors the WHP action plan (with or without service provider assistance) around three key intervention areas (policy, people and place or physical environment). Implementation of the WHP is designed to lead to changes in the work environment, work culture, work productivity and individual level health outcomes. GHaW offers limited financial support, to assist businesses to implement a WHP. This support depends on the size of the business and, to encourage participation, is awarded once at least half of the business’ employees complete brief health checks. Service providers receive payment for services rendered at each stage of delivery (e.g., conducting brief health checks or delivering action plans).

### Study design and recruitment

A concurrent nested mixed-method design was used to evaluate the implementation of the GHaW program (Table 1) [38]. The mixed-method approach follows recommended approaches for evaluating complex programs [39]. The evaluation consisted of a quasi-experimental survey and nested qualitative data collection (described separately below).

**Table 1:** data collection measures

	Baseline		6 months		12 months	
	GHaW	control	GHaW	control	GHaW	control
Quantitative						
Outcome measures						
Work culture	√	√	√	√	√	√
Work productivity			√	√	√	√
Process measures						
Satisfaction with the program			√		√	
Leadership commitment	√	√	√	√	√	√
Health beliefs	√	√	√	√	√	√
Stage of the program			√		√	
WHP	√	√	√	√	√	√
Business characteristics	√	√				
Qualitative						
Interviews with service providers					√	
Interviews with business key contacts					√	
Focus groups with employees					√	

A list of key contacts from non-government businesses registered for the program when it launched in 2014, were contacted by the authors by phone and email and invited to participate in an online survey (Additional file 1). A control group of businesses not registered in GHaW at baseline were recruited from a market research panel. Key contacts in both the intervention and control businesses were identified as having some responsibility for workplace health. Participating businesses were surveyed at baseline (T1), six months’ (T2) and 12 months’ (T3) follow-up to assess implementation and short-term business-level effects, which might be evident within 12 months.

We used qualitative methods to investigate factors influencing engagement and participation in GHaW and to identify how the program elements and mechanisms worked in practice. The qualitative research was inductive and aimed to explore the outcomes of the program, the processes and mechanisms guiding the various levels of implementation, and the interactions between program recipients (businesses and workers) and program implementers (service providers). This allowed for emergent processes and interactions to be identified, as the nature of complex programs means that some element of uncertainty will continue even into the implementation stage.

A theoretical framework for the qualitative evaluation was not imposed, but the approach relies in part on interactionism theory [40]. However in aiming to understand the experiences of the program from the perspective of the various groups (service provider, business level and employees), we also took a phenomenological approach by gathering experiences within the context of a pragmatic complex program evaluation framework [41].

A subset of key contacts from registered businesses (stratified by business size and geography to include businesses in regional and metropolitan areas) were randomly selected to participate in a face-to-face semi-structured interview at T3 at their workplace (*n* = 11). The contact selected male and female employees to participate focus groups undertaken in the workplace (*n* = 3 groups of 4–5 participants). Focus groups did not include participants in a direct line management. Service providers engaged by the government to support implementation of the program were also interviewed about their experience (*n* = 9). Service providers were businesses qualified in workplace health services. They included staff from a mixture of backgrounds (for example, nursing, exercise science, psychology, and dietetics).

In order to maintain confidentiality and minimise bias, participants were informed that their individual responses would not be shared with program coordinators or service providers (in the case of business contacts). Written consent was obtained from all participants. All interviews were conducted by researchers with interviewing experience (authors MC, AG, EG).

### Quantitative measures

#### Exposure

The primary exposure variable was a dichotomous indicator of registration into GHaW at baseline (intervention group) versus not registering (control group). Second, a graded measure of exposure (dose received) was based on progress through the program cycle (Fig. 1), as registration alone is unlikely to lead to businesses adopting the WHP.

#### Outcomes

Primary short-term outcomes were assessed in terms of changes in perceptions of workplace culture and work productivity (“business impacts”) (See Table 2). These included effects that might occur within 12 months, and therefore focused on changes in perceptions and attitudes that lead to behaviour change [42]. Work culture measures included perceptions of *the workplace as promoting healthy behaviour and health promotion and to what degree it was open to change* on a five-point scale (from strongly disagree to strongly agree). Five-point agreement scales were also used to assess perceptions of work productivity (*absenteeism due to sickness or injury; staff retention; and productivity and satisfaction with the workplace*).

**Table 2:** Quantitative sample characteristics (at baseline, 6 months and 12 months)

Variable		Baseline (T1)		6 months (T2)		12 months (T3)	
		GHaW	Control	GHaW	Control	GHaW	Control
Total sample (n)		241	403	94	295	56	227
Workplace health program (WHP)^a^	GHaW Other WHP No WHP/ unsure	n/a 82 (34.0) 159 (66.0)	n/a 122 (30.3) 281 (69.7)	64 (68.1) 10 (10.6) 20 (21.3)	21 (7.1) 32 (10.9) 242 (82.0)	29 (51.8) 8 (14.3) 19 (33.9)	26 (11.5) 23 (10.1) 178 (78.4)
Business Size	Small < 20 Medium (20–199) Large (200+)	75 (31.4) 120 (50.2) 44 (18.4)	201 (49.9) 124 (30.8) 78 (19.4)	27 (29.4) 48 (52.2) 17 (18.5)	148 (54.0) 85 (31.0) 41 (15.0)	19 (34.5) 28 (50.9) 8 (14.6)	110 (54.7) 65 (32.3) 26 (12.9)
Industry							
Finance, insurance, scientific, technical Public admin, safety, media & comms, admin support, real estate Education, healthcare, social, arts, recreation Transport, postal, warehouse, wholesale Accommodation, food service, retail Agriculture, forestry, fishing, manufacture, construction Mining, electricity, gas, water & waste Other services		34 (13.9) 28 (11.5) 55 (22.5) 36 (14.8) 47 (19.3) 33 (13.5) 4 (1.6) 7 (2.9)	74 (18.4) 59 (14.6) 96 (23.8) 28 (7.0) 63 (15.6) 61 (15.1) 9 (2.2) 13 (3.2)	5 (5.4) 13 (14.1) 25 (27.2) 16 (17.4) 19 (20.7) 10 (10.9) 1 (1.1) 3 (3.3)	48 (17.5) 338 (13.9) 62 (22.6) 18 (6.6) 46 (16.8) 48 (17.5) 4 (1.5) 10 (3.7)	7(12.7) 10 (18.2) 15 (27.3) 5 (9.1) 10 (18.2) 6 (10.9) 1 (1.8) 1 (1.8)	39 (19.4) 31 (15.4) 42 (20.9) 11 (5.5) 33 (16.4) 32 (15.9) 5 (2.5) 8 (4.0)
Stage within the GHaW program^b^	Not started Stage 1–3 Stage 4–7	241 (100)	n/a	33 (35.1) 28 (29.8) 33 (35.1)	n/a	29 (51.8) 6 (10.7) 21 (37.5)	n/a
Work culture							
People at my workplace are generally very healthy	Agree^c^	89 (36.9)	256 (63.5)	44 (50.6)	179 (65.3)	28 (52.8)	131 (65.2)
People at my workplace rarely take sick days	Agree	89 (36.9)	241 (59.8)	36 (41.4)	174 (63.5)	25 (47.2)	131 (65.2)
My workplace promotes healthy behaviours	Agree	127 (52.9)	249 (61.8)	63 (72.4)	166 (60.6)	42 (80.8)	129 (64.2)
My workplace culture is open to change	Agree	182 (75.8)	287 (71.2)	65 (74.7)	182 (66.4)	41 (77.4)	146 (72.6)
People at my workplace are willing to participate in WHP activities	Agree	156 (64.7)	203 (50.4)	62 (71.3)	132 (48.2)	32 (60.4)	110 (54.7)
Most people at my workplace could take time out of the work day to participate in a group-based program	Agree	86 (35.8)	155 (38.5)	35 (40.2)	102 (37.2)	19 (35.9)	69 (34.3)

^a^Those who indicated GHaW WHP are removed from the comparison group frequencies for all other variables at 6 months and 12 months

^b^Stage 1–3 include signing a commitment to the program, completing a workplace review and conducting the brief health check. Stages 4–7 include developing an action plan, implementing the plan, monitoring the plan or starting to implement another WHP priority area

^c^ n(%) agree vs disagree/unsure

#### Covariates

Possible mediating factors of WHP implementation (“implementation factors”) included organisational factors (business size, industry and mode of program delivery (service provider or DIY)), health promotion beliefs, support from senior leadership and satisfaction with the program. Business industry was determined by Australian and New Zealand Industry Classification as one of seven categories, and dichotomised into professional and service/industrial businesses [43]. Health promotion beliefs and leadership items were five-item agreement scales adapted from Hannon 2017 [42]. Satisfaction was assessed in terms of usefulness of the program components in setting up the workplace health program. This included the usefulness (categorised as extremely useful, quite useful, moderately useful, not so useful and not useful at all) of the brief health check, service provider, and the online resources. Program impacts were also framed in terms of whether business contacts felt that the program was implemented as planned (yes completely/somewhat, no/unsure) and if the program had its intended impacts (yes completely/somewhat, no/unsure).

### Qualitative components

Interviews with key contacts (average duration 52 min) were used to collect data on how businesses implemented the program and explored their perceptions about program registration and implementation processes and perceived impacts of the program. Focus groups with employees (average duration 58 min) were conducted to understand what employees thought of the program after it was implemented, and explored awareness of GHaW, changes in work culture and experience of participating in the BHCs and any WHP interventions. Interviews with service providers (average duration 62 min) were used to understand business engagement and use of the program from the service provider perspective and explored provider impressions of the program and perceptions about how workplaces engaged with the various stages of implementation. These interviews also explored their own involvement and engagement with the program (Additional file 2 for discussion guides).

### Data analysis

Six and 12 month follow-up impacts were analysed first and then both quantitative and qualitative data were used to understand the implementation process by which outcomes arose.

#### Quantitative analysis

During the study a number of businesses in the control group reported taking up the GHaW WHP after baseline assessment (*n* = 21, 7.1% T2; and *n* = 26, 11.5% T3). To avoid contamination, these businesses were removed from the control group for data analysis. Scales were developed to assess work culture, health promotion beliefs and leadership items using principal factor analysis with orthogonal rotation; factor items with a loading > 0.3 were retained. Scales were developed around health beliefs (5 item, Cronbach α = 0.887) and leadership (5 items, α = 0.885). Items pertaining to the workplace culture loaded across three factors (loading ~ 0.5 across multiple factors) and so were kept as single outcomes (see Additional file 3 for full details).

Changes in business outcomes were analysed as follows: Workplace culture items were compared over time within and between groups using two-level binary mixed effects logistic regression models. Outcomes were dichotomised based on agreement (strongly agree/agree vs disagree/strongly disagree/neutral). The model included random intercepts for responses over time nested within key contacts. Fixed effects were included for group, time and group by time interaction. Business size was included as a covariate given baseline differences [36]. A sensitivity analysis treating program engagement (dose received) as the exposure measure was conducted. Workplace perceived productivity outcomes were assessed at T2 and T3. Frequencies of productivity outcomes were calculated but differences between intervention and control groups were not compared due to the inability to control for potential confounding factors [44].

Implementation factors were analysed as follows: business-level engagement in, and adoption of, the program was compared across the range of mediating factors. Program engagement was defined as being at least at stage 1 of the program cycle and adoption as completing at least stage 3. Binary univariate outcomes were modelled by logistic regression using Fisher’s exact test to reduce small sample biases. Frequencies are reported for program satisfaction related variables. Stata version 14 (StataCorp LP, College Station, TX) was used for statistical analyses.

#### Qualitative analysis

Interviews were transcribed verbatim and checked for accuracy. First cycle data coding was carried out independently by two authors (MC and EG). A deductive process was applied to investigate the implementation process described in the quantitative data [45]. An inductive approach then explored nuances and emerging constructs from the interactions between the various program elements and individual groups. A third author (AG) reviewed the coding process for validation. The authors met to discuss themes identified and then interpret contextual meaning. Participant checking of the qualitative themes was not conducted given the time pressures described by participants. NVivo 11 software (QSR International Pty Ltd. Version 10, 2014) was used to code data.

## Results

The quantitative results are presented below, followed by the qualitative which provide potential explanations for the quantitative results.

### Quantitative findings

**Fig. 2:**
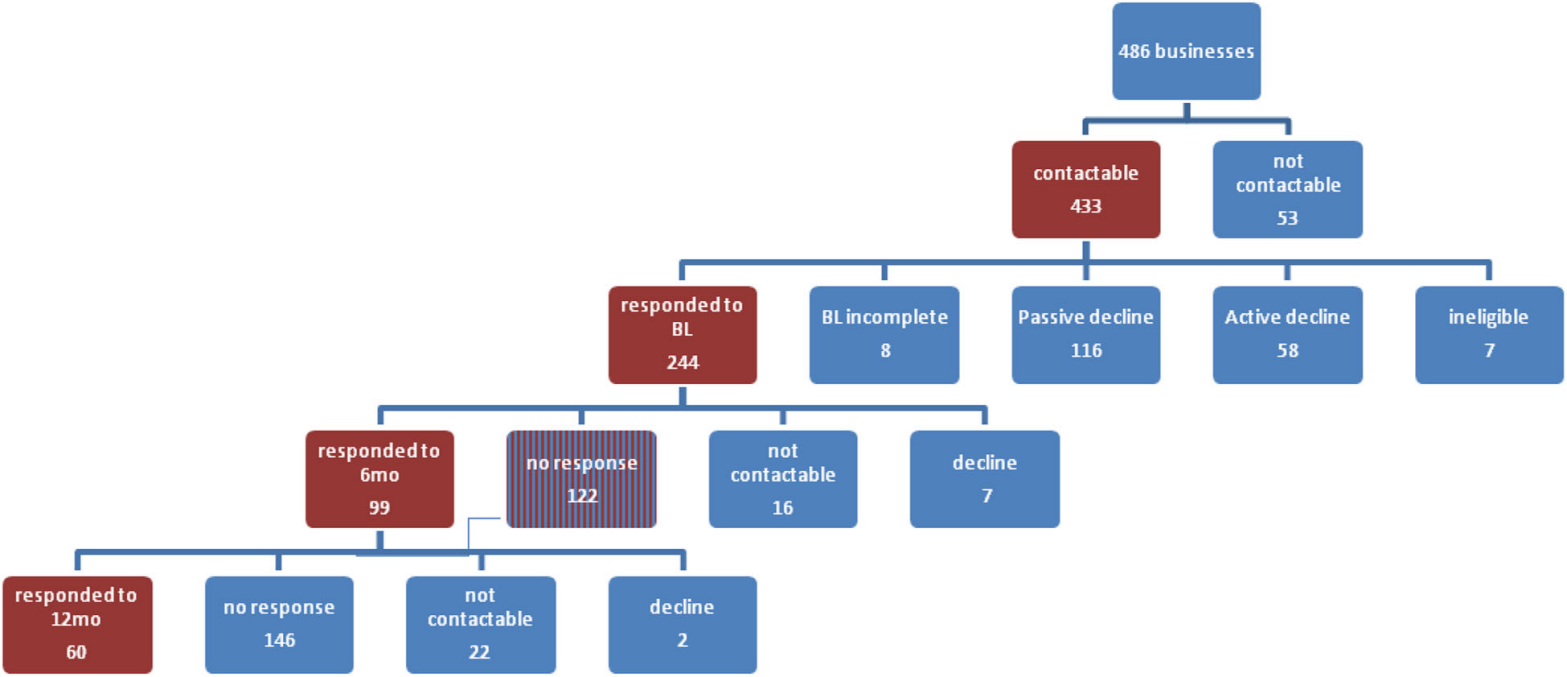
GHaW survey response flow chart. Legend: ineligible at baseline included 5 duplicate businesses; those responding to the baseline (BL) survey who also provided contact details were invited to participate in the follow-up; those not responding to 6mo survey (*n* = 122) were reinvited at the 12mo survey

In 2014 when GHaW was launched there were 696,991 private and public businesses operating in NSW [3]. At baseline (T1), 244 (50.2%) business contacts who registered for the GHaW program in 2014 and were contactable agreed to participate in the survey (Fig. 2). The survey was repeated at 6 months (T2; *n* = 94) and at 12 months (T3; *n* = 56) (Table 2). The majority of intervention group businesses (*n* = 64; 68.1%) responding at T2 indicated having adopted the GHaW program. In terms of the program cycle of the 94 business contacts surveyed at T2, 35.1% reported they had developed a WHP action plan; 29.8% had conducted just the initial stage of brief health checks; and 35.1% indicated they had not yet started the program (items are not mutually exclusive). As fewer businesses in the GHaW sample were retained at T3 (*n* = 29; 51.8%; *p* = 0.047), the results presented focus primarily on T2 findings.

#### Workplace culture

Comparison of workplace culture perceptions between the GHaW and control groups are shown in Table 3. At T1, the intervention group rated their workplace culture more negatively than the control group in a number of areas, including a lower agreement that people in their workplace are healthy (OR (95%CI): 0.19(0.11–0.35)), rarely take sick leave (OR:0.28(0.15–0.50)) and the workplace promotes healthy behaviours (OR:0.55(0.32–0.94)). In contrast, the GHaW group were more likely to report employee willingness to participate in WHP activities in their business (OR:2.52 (1.50–4.22)).

**Table 3:** Multilevel logistic regression analysis of change in perceptions about work environment and organisational culture

	Intervention effect at each time point (Control group is reference: OR = 1.0)			∆ between waves		Time x group effect
	Baseline (T1)	6 months (T2)	12 months (T3)	GHaW	Control	
Level of agreement (agree/strongly agree)	OR(95%CI)	OR(95%CI)	OR(95%CI)	P	P	P
People at my workplace are generally very healthy	0.19 (0.11–0.35)	0.45 (0.20–0.99)	0.56 (0.21–1.49)	0.013	0.946	0.045
People at my workplace rarely take sick days	0.28 (0.15–0.50)	0.32 (0.14–0.74)	0.34 (0.12–0.94)	0.470	0.528	0.897
My workplace promotes healthy behaviours	0.55 (0.32–0.94)	1.71 (0.76–3.86)	2.64 (0.89–7.84)	0.0003	0.618	0.004
My workplace culture is open to change	1.65 (0.92–2.98)	1.70 (0.74–3.88)	1.29 (0.45–3.66)	0.647	0.122	0.887
People at my workplace are willing to participate in worksite health promotion activities	2.76 (1.62–4.68)	3.75 (1.72–8.17)	1.22 (0.49–3.04)	0.271	0.340	0.118
Most people at my workplace could take time out of the work day to participate in a group-based program	0.89 (0.56–1.41)	1.17 (0.60–2.30)	0.91 (0.38–2.14)	0.563	0.710	0.749

Notes: Analyses adjust for size of business. Reference category is disagree/strongly disagree/neutral. *OR* odds ratio

Statistically significant improvements were observed over time in the intervention group for perceptions that people in the workplace were healthy (*p* = 0.013) and that the workplace promotes healthy behaviours (*p* < 0.01). The control group did not change. Willingness to participate in workplace health promotion remained higher in the intervention than the control group over time but did not change significantly (*p* = 0.27), suggesting perceptions were sustained. The remaining workplace culture items did not change over the short-term in either group.

#### Work productivity

In terms of perceptions of work productivity, nearly half (*n* = 31; 48.4%) of businesses in the intervention sample who stated they had a GHaW program at T2 agreed that the program had an impact on increasing worker engagement/satisfaction. A third agreed that it had increased worker productivity (*n* = 20, 31.3%). Few businesses indicated an impact on reducing sick leave or an increase in worker retention (both: *n* = 11, 17.2%).

#### Implementation factors

Logistic regression analysis of organisational factors associated with whether registered workplaces started the program cycle (vs not starting) found neither business size, industry, nor mode of delivery for brief health check or WHP support to be statistically significant (all *p* > 0.1). Factors relating to readiness to change, such as supportive leadership, were also not significant (*p* = 0.60), although health beliefs (in favour of workplace health promotion) neared statistical significance (OR:2.26 (0.91–6.07); *p* = 0.081).

In terms of satisfaction with the program: 63 business contacts (67%) rated the helpfulness of the brief health check. Of these, 52 (82.5%) stated it was extremely to moderately useful; only 11 (17.5%) said it was not useful. There was no significant difference between whether the business had used the online or service provider-assisted brief health check in terms of its usefulness. The helpfulness of the service provider was rated by almost all of those who used a service provider (*n* = 36; 94.7%). Of these 36 businesses, 4 stated that the service provider had made no contact with them by T2, while 26 rated the service provider as extremely to moderately helpful. The online resources were rated by only 26 (27.7%) of businesses, of which 3 stated the resource was not useful. These results are an indication of usefulness however may not be representative of the whole sample. Most businesses which had adopted GHaW by T2 reported that the program proceeded as planned (26 of 32 businesses) and had its intended impact (29 of 33 businesses).

### Qualitative insights

Interviews with the subsample of business key contacts (*n* = 11), employee focus groups (n = 3) and service providers (*n* = 9) provide further insight on the implementation process. Key contacts were from ten businesses which had completed the brief health check and four which had also implemented a WHP. All businesses interviewed had requested service provider support (Additional file 4 for business characteristics). Factors influencing implementation of the program were categorised as pertaining to either the organisation or program (including the program characteristics and delivery). Table 4 provides a summary of these themes.

**Table 4:** Overview of constructs contributing to the implementation of the get healthy at work program

Level	construct
Organisational factors	Previous experience of WHPs
	Motivation to adopt the program
	Supportive leadership
	Business priorities
	Organisational structures
Program characteristics	Government delivered program
	Financial incentives
	Health priority focus
	Sequential program cycle
	Sustainable action plans
Program delivery	Service provider support
	Information technologies and processes
	Communication
	Service capacity

#### Organisational factors

Two central overlapping factors appeared to underpin the implementation of the program across workplaces at the organisational level: business size and previous experience implementing WHPs. Business size and experience ran parallel in that smaller businesses had little previous experience of WHPs while larger businesses generally had previous experience developing WHPs, and therefore were more likely to select program elements. Smaller businesses may have felt ready to implement a WHP but were less familiar with the process of a WHP, or what it involved. They were therefore more in need of assistance: “*It was all new to me and I really didn’t understand what the process was”* (business, small).

Engagement with GHaW appeared predominantly driven by the business management’s motivation to improve employees’ health, irrespective of business size or WHP experience. *“We noticed the guys buy a lot of chips and pies and things like that at lunch time. So what we were thinking of doing was trying to put a healthy eating program in place”* (business, medium). Yet further adoption of the program from the business’s end depended on leadership involvement or endorsement and alignment of the program with business priorities. Leadership actions, such as allowing WHP participation during work hours, and health beliefs, such as feeling a business has a role in employee health, had potential to drive the program. Two of the focus groups spoke of the difficulty getting things moving because of a lack of champion or push from senior level.

Small businesses with competing priorities struggled more in this respect. Organisational priorities and WHP implementation did not always align. Service providers perceived smaller businesses were unlikely to reach the implementation stage because employee wellbeing was not a high priority. Some business contacts stated competing priorities as a reason for their slow progress through the program cycle: *“It’s probably not as top priority as for me as so many other things I’ve got to do at the moment, so that’s another issue I think*” (business, small).

Organisational structures presented other challenges to implementation such as logistical challenges (if multi-site or if trading outside NSW) or administrative challenges (in the case of takeovers and mergers). For businesses with national or international management, WHP implementation at a local level could require a lengthy process of approvals and depend upon what is already in place nationally/ internationally. As observed by one service provider, “Every stage they [participating businesses] have to go through a level of approval before they’ll do it and it seems to be a long drawn out process, so no. We haven’t noticed that many get through”.

#### Program characteristics

A key reason for most businesses and service providers engaging with the program was the government sponsorship of the program. The Government was viewed as a trustworthy, reputable provider. “*It’s good to be seen as a company … as this organisation to be aligned with the government and supporting peoples’ health”* (service provider). This was also important to one focus group: employees were favourable toward government involvement in workplace health, explaining it made sense given the amount of time people spend in the workplace and government’s position of influence over businesses’ approaches to employees’ health.

The financial incentive to support implementation of a WHP was a major attraction and factor in supporting small businesses implementing the program. “*This is something we’ve had discussions about in different groups is small businesses ... struggling to survive most the time let alone thinking about putting more money into programmes... to support their workers”* (business, small). Perspectives on the cost of running the program changed from being a barrier to an expense justified by the observed benefits, but for those businesses who stated they were unable to achieve the participation requirements in order to receive the incentive, this became a discouragement. “*We planned to get to 50 so that we would get the report… But because we didn’t get to 50, we didn’t get the report...there was no tailored programme”* (business, medium).

The steps within the GHaW program cycle were poorly understood, with a few business contacts perceiving the brief health check to be the entirety of the program. “*I guess, also, because they may have only used the program for the brief health checks. So they don’t see the big picture”* (business, large). Completion of the brief health check was essential for moving on to the next stage however for many businesses, especially for multi-site business, the logistics of organising employees to complete this step was a challenge. While the online option provided flexibility, some businesses stated that staff computer access and literacy skills could be participation barriers.

Other program characteristics seemed to challenge implementation. For example, GHaW recommends that a single health priority is the WHP focus, but the majority of businesses interviewed wanted a more encompassing “wellbeing” program. “*Everyone’s done an online health check, they’ve come up with an individual risk factor, and yet we’re not necessarily targeting that because we have to do something for everybody”* (business, small). Interviewed businesses that had implemented some WHP elements all listed both dietary and physical activity initiatives. This observation was also made by all service providers. “*It’s been difficult getting companies to get on board to do smoking cessation programmes. …they see it as, I want to be offering something to the majority of my staff. ... I want to run a nutrition workshop. I want to get a better attendance, there’ll be more people engaged with it”* (service provider). As such, WHP action plans appeared to be adopted piecemeal.

GHaW also recommends a mix of people, place and policy initiatives. Activity-based interventions (such as weekly exercise activity or gym equipment) appeared to attract low participation while policies (such as changes from unhealthy to healthy food catering options implemented by many of the groups) appeared to be more encompassing.

#### Program delivery factors

Program delivery processes impeded implementation in a number of ways. Business contacts reported time delays between when businesses registered and when contacted by the service provider. This had implications on their enthusiasm for the program and momentum along the program cycle. “*I was still very open to it, but I think I was a bit more like, “There’s no urgency from that side, so there’s no urgency from my side”,…I think after I signed up for it, …somebody rang me …we’ve talked about, like why I signed up… I didn’t hear anything more about that”* (business, small).

The service providers’ delay in contacting businesses were reasoned as being due to initial administrative processes (such as manual processing of brief health checks), and challenges with the website not being fully operational and delays in their accessing of the online portal.. “*This would be easy now that they’ve gone online. But previously the sending of the questionnaires to a certain document management group who would scan the things and put it up online into the profile was tedious and made things difficult”* (service provider). Communication between service providers and the GHaW staff was not always effective; further, service providers all reported difficulties when escalating issues and that this often involved two separate government departments. Communication was also unclear from the business’s perspective, not only about how the program worked, but also how the service provider was involved. “*It was never very clear about how the system works, I really didn’t understand how they* [service provider] *plugged into Get Healthy at Work. Those sort of things that you had these separate providers and it wasn’t terribly clear*” (business, small).

Service capacity was also discussed by the service providers as a complication. Some mentioned logistical issues of agreeing to contact a business located in a certain part of the state, only to find that the business was primarily based in another location that might be harder to reach. Many felt that the program ran a high operational cost, and so a balance between investment and revenue generation was felt to be unattained. “*We’re just not even getting to the point where we’re delivering an action plan so we can offer those services. Because we’re not actually allowed to offer them until the action plan’s completed”* (service provider). This appeared to affect their commitment, as a group, to the program.

(Further details about key factors are described in Additional file 5).

## Discussion

One of the main challenges to the dissemination of health promotion programs in the workplace is the lack of evidence on the effectiveness of interventions within the real-world implementation context [46]. This evaluation sought to assess the business-level impacts and factors supporting implementation of a state-wide comprehensive WHP which included a focus on health education, supportive environments, policies to integrate the program into the workplace, resources and implementation support [31]. Implementation of WHPs at the business-level will determine individual worker-level outcomes. For this reason the evaluation focused specifically on the implementation process and the impact at the business-level. The evaluation research conducted with employees was used to gather information about the implementation process across individual businesses, rather than to evaluate worker-level outcomes, which would be unlikely to change over the short-term intervention period. This means we were concerned with assessing change at the organisational level, including organisational culture and climate [47]. These need to be assessed prior to any impact evaluation of employee-level outcomes as such context-free evaluation will provide limited and potentially misleading conclusions about a program [24]. The evaluation also helps to identify the obstacles and challenges involved in implementing a state-wide health promotion program which can be used to improve future program delivery.

Some positive impacts were observed which support GHaW as a vehicle for enabling NCD prevention across the workforce population. Specifically, survey findings reveal that while GHaW participants reported initially a much lower perception of their workplace as healthy or health promoting at registration, this improved significantly over the course of the evaluation to be well above control group perceptions (which remained unchanged). This suggests that interaction with the GHaW program had a positive impact on the business contacts’ perceptions of health promotion in their workplace. Poor perceptions of health promotion in their workplace at baseline may have been an essential reason why this group of businesses first registered for the program in the first place [48–50]. The intervention group retained a higher willingness to participate in WHP activities. Together, these results suggest some level of organisational change occurred, an essential critical condition for effective implementation and success of workplace health promotion initiatives [18, 51]. Improvements in workplace culture did not lead to improvements in perceived work productivity within the evaluation period. However it may be too early to gauge evidence of impact because of the lag in engagement: nearly a third of businesses had not yet started the program cycle by T3 and many others were still only at the initial pre-implementation stage of conducting brief health checks in the workplace. No specific organisational characteristic in the quantitative analyses explained why most businesses had not yet proceeded further along the program cycle.

Investigation of the implementation processes through the qualitative interviews with business contacts and service providers and the focus groups with employees revealed a number of factors contributing to the program outcomes observed; these include both business level factors and program and delivery factors. A recent synthesis of qualitative studies across all phases of WHP development identified six areas that facilitate or hinder WHP implementation. Three identified factors (which we also assessed) related to the intervention, the implementer and organisational level; the other three factors included the participant level, the planning phase, and evaluation methodology [47]. Our qualitative analysis similarly identified organisational characteristics influencing program implementation, including business size and structures and experience with WHPs. For example, larger businesses were found to ‘pick and choose’ components of GHaW, like the brief health check, according to their workplace needs and may have used GHaW as part of a broader, internally-developed and pre-existing WHP.

Unfamiliarity with WHPs in general was a major determining factor for how smaller businesses interacted with the GHaW program and in their initial struggle to connect their desire to change their workplaces with the ability to navigate the steps involved. Small businesses make up 97.5% of businesses in Australia [52] and are therefore an important group to equip to promote health. Smaller businesses, lacking corporate structure and personnel, must balance an interest in health promotion with core business priorities, and this was evident in the qualitative analysis. Despite financial incentives being a major facilitator for small businesses to adopt GHaW, changes may be required to develop these incentives to make it easier for businesses to implement WHPs alongside other business priorities. Organisational readiness for change and supportive senior leadership were also identified as influential factors in the qualitative data and elsewhere [47]. Senior leadership support is frequently reported as a facilitator of WHP implementation and effectiveness within a workplace [53]. Leadership commitment, measured quantitatively in this study, was not a statistically significant factor, possibly due to the size of this sample of participants who had implemented the WHP.

Government may play a central role in enabling prioritisation and incentivising health promotion in the workplace [54]. We found that while small businesses are motivated to improve the health of their employees, they have limited tools and knowledge to implement WHPs. Government delivery and resource support were strong reasons businesses registered in the program. A lack of resources has often been reported as a barrier to WHP implementation [47, 53]. GHaW’s financial incentive was particularly important to smaller businesses; however many had difficulty completing the brief health checks, a requirement for eligibility for receiving the financial benefit. A mismatch between what businesses desired from a WHP and how GHaW was offered led to variable implementation. Rojatz et al. [47] also identified appropriateness of the intervention as central to business level implementation. We found that ‘wellness’ programs which include healthy eating and physical activity initiatives are preferred by businesses, and there was less interest in programs which specifically target alcohol consumption, active travel or smoking cessation, issues which may not encompass all employees. While GHaW can be tailored to a variety of employee health priority needs, the expressed preference points to allowing selection of a suite of priorities to suit business needs [55].

Program delivery factors were also identified as potential barriers to implementation. Technical issues and delays in the operation of the online portal, complicated administrative processes and unclear communication were reasons given for delays in businesses receiving service provider support in the early stages of the program. This may be a key reason for why less than one third of businesses sampled had developed a WHP after 12 months. Quality improvements to processes, for example automation of some of the service provider administrative tasks that were occurring over the course of the evaluation, and clearer communication channels are likely to mitigate many of these issues for future participants [48]. In order to improve long-term impact and sustainability of health promotion in workplaces, viability of the program as a business proposition for service providers may also need consideration. Partly this may be through better communication of the role of the service provider, yet it may also involve other strategies given the demand for service provider assistance to develop tailored WHPs.

Complex WHP programs like GHaW require multi-level evaluation methods to determine what worked, how it worked and what mechanisms enabled the program to work in this way [56]. In one sense, it might appear that every group involved in the implementation attributes delay or failure to an external source. Yet the reality is that delivery of programs at-scale is often complicated and implementation is challenged by many discordant factors combining to influence outcomes in nonlinear and often unpredictable ways [57]. Understanding this complexity is important for building the evidence to inform policy and practice [23].

The complexity of WHP does not mean that the evaluation needs to be complex [26], however there needs to be some understanding of the mechanisms or processes and context which produce the outcomes observed [58, 59]. Essential in any complex program evaluation is an evaluation of the implementation process. Unfortunately, evaluation of the implementation process is still rarely applied to WHP programs in practice [53]. Implementation context, in particular, is central to understanding outcomes, improving the reach and uptake of health promotion programs and ensuring their generalisability.

A major strength of the study was that it employed a mixed method approach, which integrated quantitative and qualitative data sources. The quasi-experimental longitudinal analysis allowed individual business responses assessed over time, adjusting for underlying trends in the control sample to account for baseline differences. Additionally, using qualitative data from different perspectives and points in the system (i.e., service providers, business key contacts and employees) further enriched the analysis of the quasi-experiment. Moreover, evaluations embedded into program delivery can facilitate continuous improvement of programs to optimise program engagement and outcomes. Ongoing program evaluation, which includes feedback loops in WHP design for ongoing refinement and course-correction, is an important practice for an effective program [31]. The evaluation of the GHaW program [36, 60] has led to a number of service delivery enhancements. Some of the modifications include simplification of the program for small businesses, increased funding for service providers to engage with businesses at early stages of the program, and regular electronic messaging to businesses to provide program updates. Redevelopment of the GHaW web portal is currently underway to address delivery issues raised by business contacts and service providers.

One of the major challenges of evaluating health promotion programs in practice is determining population impact. The reach of the GHaW program when it was first launched was limited if we were to consider just the initial sample in its first year as a proportion of all businesses in the State (which was less than 1%). A population dose of < 2% could be considered low impact, while a dose above 5% could be considered both significant and measureable [61]. However these estimates are also arbitrary [61], and in the case of WHPs, must also account for businesses size and other factors: larger businesses reach higher numbers of the workforce population, but smaller businesses often include the harder to reach workforce. [46].

A limitation of the study was the rate of attrition in the evaluation sample; particularly at T3 amongst those who had reached the stage of adopting a WHP action plan. This attrition also highlights the challenges of evaluating WHPs in practice. Within the course of a year, businesses may expand or fold or change operating structures as revealed in the qualitative study and response rate of the survey. This can have a profound impact on evaluation. Tracking changes in the intervention group against a control sample, representative of the wider population of businesses, strengthens the study by accounting for secular trends which may be occurring over time [44] and consequently the ability to make causal inferences about the program. This was a quasi-experiment. The rigour of the study design may have been strengthened were a natural experiment approach used to assess exposure to the GHaW program, i.e., before the intervention group was established, however this would add substantially to the cost and time demand.

The study relied on subjective measures of productivity and businesses’ interpretation of how far into the program cycle they progressed as a proxy for objective measures. Subjective responses were based on the opinions of the business contact who is likely to have a vested interest in the WHP and its impact. As noted earlier, this evaluation prioritised evaluating implementation at the business level over the employee level, assuming implementation at the business level leads to employee level impacts. Focus groups were used to develop an understanding of the impact as viewed at the employee level, however few businesses contacted felt they had the time or the logistical ability to enable employees to participate. The evidence obtained from these focus groups is therefore incomplete and should be treated with caution. This evaluation was conducted with businesses that were early adopters of the GHaW program and the findings therefore may not be representative of businesses in NSW. Nevertheless, the evaluation provides insights into the potential effectiveness of GHaW, as well as the complexities of implementing WHPs.

## Conclusion

This study sought to assess the implementation and short-term impacts of an at-scale state-wide WHP. Evaluation of the implementation process revealed some issues in program delivery and in the characteristics of the program that were not matched with what businesses want in a WHP, which may have contributed to why so few businesses had reached a stage of developing workplace action plans. The findings suggest businesses may benefit from earlier involvement by service providers, clearer communication and a supportive system to enable businesses to develop programs tailored to their needs and best practice.

## Additional files

Additional file 1: Quantitative business key contact survey. (DOC 61 kb)

Additional file 2: Qualitative data key stakeholder interview discussion guides. (DOC 40 kb)

Additional file 3: **Table S1.** Quantitative survey items factor analysis and scale results. (DOC 52 kb)

Additional file 4: **Table S2.** Qualitative research sample characteristics of participating businesses. (DOC 36 kb)

Additional file 5: **Table S3.** Detailed qualitative data findings. (DOC 53 kb)

## Abbreviations

DIY: Do-it-yourself
FG: Focus group
GHaW: Get Healthy at Work
SP: Service Provider
WHP: work health programs
