# Supplementary material for: Evaluation of Get Healthy at Work, a state-wide workplace health promotion program in Australia
Source: BMC Public Health. 2019 Feb 13;19:183. doi: 10.1186/s12889-019-6493-y (PMC6373144; doi:10.1186/s12889-019-6493-y)
Supplement: Supplementary file 1 — Quantitative business key contact survey. (DOC 61 kb) [file 12889_2019_6493_MOESM1_ESM.doc]

**Get Healthy at Work - Worksite Key Contact survey**

Workplace health promotion programs are a coordinated set of activities implemented at the workplace that address individual health (people), the workplace environment (place), and policy, designed to impact positively on the health of all workers.

**New workplace health promotion programs**

1. Tick the following box if you consent to participate in this survey

⬜

1. In the past 6 months, have you implemented any workplace health promotion programs?

Yes, Get Healthy at Work ⬜

Yes, other program ⬜

No (skip to Q11) ⬜

Not sure (skip to Q11) ⬜

1. If yes, did the workplace health promotion program(s) include a focus on any of the following? (Tick all that apply)

Active travel⬜

Alcohol⬜

Healthy eating ⬜

Healthy weight ⬜

Mental health⬜

Physical activity ⬜

Smoking cessation ⬜

Other, specify________________⬜

1. If yes, what was the target area for the workplace health promotion program?

(Tick all that apply)

Individual workers ⬜

Workplace policies ⬜

Workplace physical environment ⬜

Other, specify________________⬜

1. If yes, how much do you estimate this workplace health promotion program will cost per year to implement?

Nothing ⬜

<$10,000 ⬜

$10,000-<$100,000 ⬜

$100,000+ ⬜

Not sure⬜

1. If yes, how many workers do you think know about the workplace health promotion program?

All staff ⬜

Most staff ⬜

Some staff ⬜

A few staff ⬜

No staff ⬜

1. Did implementing this workplace health promotion program proceed as planned?

Yes, completely ⬜

Yes, somewhat ⬜

No ⬜

Not sure ⬜

1. Was the cost of implementing this workplace health promotion program more or less than what you expected?

More than expected ⬜

Same as expected ⬜

Less than expected ⬜

Not sure ⬜

1. Do you think this workplace health promotion program had its intended impacts?

Yes, completely ⬜

Yes, somewhat ⬜

No ⬜

Not sure⬜

1. Do you think this workplace health promotion program had an impact on: (Tick all that apply)

Reducing sick leave ⬜

Reducing incidence of workplace injury ⬜

Increasing worker engagement/satisfaction ⬜

Increasing worker retention ⬜

Increasing worker productivity ⬜

Other, specify____________ ⬜

**Workplace Health Promotion**

1. For the following question please indicate your level of agreement with each statement.

|  | Strongly disagree | Disagree | Neither agree nor disagree | Agree | Strongly agree |
| --- | --- | --- | --- | --- | --- |
| People at my workplace are generally very healthy. | 🞏 | 🞏 | 🞏 | 🞏 | 🞏 |
| People at my workplace rarely take sick days. | 🞏 | 🞏 | 🞏 | 🞏 | 🞏 |
| My workplace promotes healthy behaviours. | 🞏 | 🞏 | 🞏 | 🞏 | 🞏 |
| My workplace culture is open to change. | 🞏 | 🞏 | 🞏 | 🞏 | 🞏 |
| Health promotion at my workplace would improve employee health. | 🞏 | 🞏 | 🞏 | 🞏 | 🞏 |
| Health promotion at my workplace can help improve employee productivity. | 🞏 | 🞏 | 🞏 | 🞏 | 🞏 |
| Health promotion at my workplace can increase staff retention. | 🞏 | 🞏 | 🞏 | 🞏 | 🞏 |
| Health promotion at my workplace can reduce the incidence of workplace injuries. | 🞏 | 🞏 | 🞏 | 🞏 | 🞏 |
| Health promotion at my workplace can help reduce sick leave. | 🞏 | 🞏 | 🞏 | 🞏 | 🞏 |
| Senior leadership at my business is willing to dedicate financial resources to worksite health promotion. | 🞏 | 🞏 | 🞏 | 🞏 | 🞏 |
| Senior leadership at my business is willing to dedicate staff time to worksite health promotion. | 🞏 | 🞏 | 🞏 | 🞏 | 🞏 |
| People at my workplace are willing to participate in worksite health promotion activities. | 🞏 | 🞏 | 🞏 | 🞏 | 🞏 |
| Most people at my workplace could take time out of the work day to participate in a group-based program, such as a walking club or weight watchers at work. | 🞏 | 🞏 | 🞏 | 🞏 | 🞏 |
| In general, senior leadership at my workplace is proactive about making changes when problems are identified. | 🞏 | 🞏 | 🞏 | 🞏 | 🞏 |
| In general, where there is agreement at my workplace that change needs to happen, there is the necessary financial support. | 🞏 | 🞏 | 🞏 | 🞏 | 🞏 |
| In general, where there is agreement at my workplace that change needs to happen, there is the necessary support in terms of staffing. | 🞏 | 🞏 | 🞏 | 🞏 | 🞏 |

***Get Healthy at Work program (GHaW participants only)***

**Workplace health program**

1. Which service delivery option did you chose for developing your workplace health program with the Get Healthy at Work service? *(Not for Brief Health Check)*

Online/Do-it-Yourself ⬜

Face to Face/Service Provider ⬜

1. Why did you choose this option?

|  |
| --- |

1. If you could do the Get Healthy at Work program over again, would you still choose this service delivery option for developing your workplace health program?

Yes ⬜

No ⬜

Not sure ⬜

1. How helpful was the service provider with setting up your workplace health program?

Extremely helpful ⬜

Quite helpful ⬜

Moderately helpful ⬜

Not so helpful ⬜

Not at all helpful ⬜

Did not use a Service Provider ⬜

1. How far along the project cycle in developing your workplace health program did you get in the past 6 months? (Tick all that apply)

Get the ball rolling ⬜

Work out needs ⬜

Brief Health checks ⬜

Prioritise health issue and develop a plan ⬜

Implement plan ⬜

Monitor and review plan ⬜

Begin a new cycle with a different health issue ⬜

**Brief Health Checks**

1. Which service delivery option did you chose for implementing worker Brief Health Checks?

Online/Do-it-Yourself ⬜

Face to Face/Service Provider ⬜

1. Why did you choose this option?

|  |
| --- |

1. If you could do the Get Healthy at Work program over again, would you still choose this service delivery option for implementing worker Brief Health Checks?

Yes ⬜

No ⬜

Not sure ⬜

1. How helpful was the Service Provider for implementing worker Brief Health Checks?

Extremely helpful ⬜

Quite helpful ⬜

Moderately helpful ⬜

Not so helpful ⬜

Not at all helpful ⬜

Did not use a Service Provider ⬜
