# Supplementary material for: Evaluation of Get Healthy at Work, a state-wide workplace health promotion program in Australia
Source: BMC Public Health. 2019 Feb 13;19:183. doi: 10.1186/s12889-019-6493-y (PMC6373144; doi:10.1186/s12889-019-6493-y)
Supplement: Supplementary file 2 — Qualitative data key stakeholder interview discussion guides. (DOC 40 kb) [file 12889_2019_6493_MOESM2_ESM.doc]

**Supplement file 2**:

**Get Healthy at Work - Key stakeholder interviews**

A) Business key contact interview

1. **Warm-up:** Participant consent obtained, Describe role in organisation; how long worked at organisation
2. **How did the KC become involved with GHaW and initial impressions of concept:** Determine initial impetus (external or own idea); was there anything that happened at the worksite that prompted initial interest; how did he/she know about GHaW; what appealed about GHaW; did the KC have any concerns initially
3. **Description of workplace culture and climate on health prior to engagement with GHaW**: General impression; differences by work group; what facilities/processes to support being healthy at work existed prior to GHaW; had the KC tried any other approaches to improving workplace health prior to GHaW
4. **Experience of initial engagement with GHaW:** How did the KC(where appropriate) find the registration process (website generally; information to be provided; options requiring decisions – delivery mode, service provider (auto or manual)); how did they may the choice between online or face-to-face for program delivery and BHC; what were their expectations of the service and did their initial impression fit with those expectations
5. **Experience of “getting the ball rolling”:** Did they use any of resources provided for this phase; How useful; describe process and experience of interactions with service provider; describe getting worksite onboard – methods/difficult/easy/what worked and what didn’t work; what/when got them to point where they decided to “go ahead”
6. **Describe and give impression of data collection, workplace health summation, selection of health priority and development of action plans:** Success in terms of participation in different data collection exercises (workplace health review, BHCs, worker survey); challenges encountered in organising these tasks; impression of their utility in selecting a health priority and to producing action plan; did it change the KC view of what the main health priority was; confidence and comfort with priority and actions chosen; what were the healthy priority and actions that were selected; with hindsight would they have chosen differently (too few/too many/not feasible)
7. **Describe implementation of action plan:** What worked and what did not work; was the plan realistic; what support did they receive from the SP and satisfaction with that support; did more or less workers participate than anticipate; did/could they adjust as things were put into place; what obstacles did they encounter and could they have been avoided; what would have assisted where actions were not able to be undertaken;
8. **Impacts on workers/workplace:** Did they notice any health benefits from the program either directly or indirectly and intended or unintended; did they perceive a change in workplace climate and culture in terms of health matters workplace – what did that look like; impressions why people did not participate (if any); do they perceive any change in sick leave/worker retention/worker engagement/satisfaction; has the existence of the program and any associated changes been used to attract future employees; have there been non-health-related corollaries (benefits or downsides; personal for KC and for workers and worksite generally);
9. **Capability/capacity to implement WHP:** How far along the project cycle in developing their workplace health promotion program did they get in the past year? Were there any stages that were more challenging than others? Do they feel capable of implementing WHP again in the future? Why or why not?
10. **Future direction**s: Do they intend to choose another health priority (and why yes or no); would they approach the task differently in terms of process or in terms of content if they could do it over again;

B) Workers focus groups

1. **Warm-up:** Participant consent obtained, Describe role in organisation; how long worked at organisation
2. **How did the workers first hear about GHaW and initial impressions of concept:** by what channel did he/she hear about GHaW and were they initially interested; what appealed about GHaW and what did not; did they think it was necessary for themselves or their co-workers; had they experienced anything similar in this or other workplaces; what degree of success did they think the program would have and why (did they think it would be supported by workers and/or management); what were the methods used to gauge or stimulate interest in the program did they think that were appropriate;
3. **Description of workplace culture and climate on health prior to engagement with GHaW**: general impression; differences by work group; what facilities/processes to support being healthy at work existed prior to GHaW;
4. **Describe and give impression of data collection (brief health check), selection of health priority and development of action plans:** what action plan development processes did the workers engage with; how useful did they think each data collection activity was generally and personally; reasons for not engaging; did they think the chosen priority was appropriate for them personally and/or for the worksite; did they think actions would achieve change (whether they actually did or did not in the end);
5. **Describe experience of implementation of action plan:** what activities did the workplace initiate; did they engage in any of the GHaW activities (why or why not); what worked and what did not work for workers personally; did it live up to expectations or exceed them; impressions why other people did/did not participate (if any);
6. **Impacts on workers/workplace:** have they experienced any health benefits from the program either directly or indirectly and intended or unintended; did they perceive a change in workplace climate and culture in terms of health matters workplace – what did that look like; have there been non-health-related corollaries;
7. **Future direction**s: would they participate in another health priority (and why yes or no); do they think those running the program should approach the task differently in terms of process or in terms of content;

C) Service provider key stakeholder interview

1. **Warm-up:** Participant consent obtained, Describe qualifications and experience in health promotion in the workplace; how many workplaces engaged with for GHaW; how many carried through until final phase
2. **How did the SP become involved with GHaW and initial impressions of concept:** describe how became involved – how heard about it, what attracted them to be involved; what did he/she think of concept; what did they think of general process (overview only – detailed opinions of resources and process to follow); had he or she been involved in similar programs previously – how was this different from previous initiatives;
3. **Workplaces engaged with and first stages of implementation**: describe workplaces the SP had contact with (industries, size, type of workers); why does he/she think those that did not follow through to the first stage drop out; is there anything that GHaW could have provided that may have prevented this; what do they think of the registration process and resources provided to business to “get the ball rolling”; is there anything that could be provided that might make it easier for the SP to get workplaces to engage; what were the key issues that determined follow-through to next stage; **Data collection:** how did they find the data collection activities in terms of informing the health priority selection; what resistance if any did they experience;
4. **Selection of health priority and development of action plan from SP perspective**: what was the range of experiences across the various workplaces in terms of health priority selection – did it match the workplace’s impression of what was important (if not why not, and how was it handled); how did the SP think their own background and skills may influence the process of health priority selection; did they feel comfortable/confident with health priorities that were not be their own particular field of expertise; how often did they get engaged to provide services outlined in the action plan; do they think the choices made in actions plans were feasible – in what circumstances were they not, and how did that come about; what were workers’ reactions to BHCs; how did they react to referrals to Quitline and GHS;
5. **Describe implementation of action plans from SP perspective:** what were the circumstances across the range of workplaces under which action plans were successfully implemented and where/why were others not successful; impressions why people did not participate (if any);
6. **Impacts on workers/workplace:** were there changes in health outcomes the SP observed; did they perceive a change in workplace climate and culture in terms of health matters in any of the workplaces – if so, what did that look like; in any of the workplaces has this experience inspired other changes in terms of health not described in the action plan; have there been non-health-related corollaries at the worksites he/she attended (benefits or downsides for workers and worksite generally); how has the SP benefitted (other than financially) from being part of GHaW (ongoing work, satisfaction, new skills, engagement with government); were the financial incentives to do the work sufficient (both in terms of the SP making a living but also in terms of giving them sufficient time to spend on the workplaces)
7. **Future directions**: did the workplaces choose another health priority (and why yes or no); would the SP be interested in continuing involvement with program; what changes do they think would be helpful to improve their own experience and/or that of workplaces and workers;
