# Supplementary material for: Evaluation of Get Healthy at Work, a state-wide workplace health promotion program in Australia
Source: BMC Public Health. 2019 Feb 13;19:183. doi: 10.1186/s12889-019-6493-y (PMC6373144; doi:10.1186/s12889-019-6493-y)
Supplement: Supplementary file 3 — Table S1. Quantitative survey items factor analysis and scale results. (DOC 52 kb) [file 12889_2019_6493_MOESM3_ESM.doc]

**Supplement file 3**:

**Table 1: Factor analysis of o**rganisational culture items and scales

| **Leadership commitment questions** | | **Factor 1** | **Factor 2** | **Factor 3** | **Cronbach α** |
| --- | --- | --- | --- | --- | --- |
| Item 1 | Senior leadership at my business is willing to dedicate financial resources to worksite health promotion | 0.809 |  |  |  |
| Item 2 | Senior leadership at my business is willing to dedicate staff time to worksite health promotion | 0.744 |  |  |  |
| Item 3 | In general, senior leadership at my workplace is proactive about making changes when problems are identified | 0.790 |  |  |  |
| Item 4 | In general, where there is agreement at my workplace that change needs to happen, there is the necessary financial support | 0.824 |  |  |  |
| Item 5 | In general, where there is agreement at my workplace that change needs to happen, there is the necessary support in terms of staffing | 0.810 |  |  |  |
| Scale reliability |  |  |  |  | 0.885 |
| **Health beliefs questions** | |  |  |  |  |
| Item 1 | Health promotion at my workplace can improve employee health |  | 0.820 |  |  |
| Item 2 | Health promotion at my workplace can help improve employee productivity |  | 0.823 |  |  |
| Item 3 | Health promotion at my workplace can increase staff retention |  | 0.822 |  |  |
| Item 4 | Health promotion at my workplace can reduce the incidence of workplace injuries |  | 0.771 |  |  |
| Item 5 | Health promotion at my workplace can help reduce sick leave |  | 0.791 |  |  |
| Scale reliability |  |  |  |  | 0.887 |
| **Work culture questions** | |  |  |  |  |
| Item 1 | People at my workplace are generally very healthy |  |  | 0.813 |  |
| Item 2 | People at my workplace rarely take sick days |  |  | 0.742 |  |
| Item 3 | My workplace promotes healthy behaviours |  | 0.431 | 0.544 |  |
| Item 4 | My workplace culture is open to change |  | 0.511 | 0.518 |  |
| Item 5 | People at my workplace are willing to participate in worksite health promotion activities | 0.487 |  | 0.452 |  |
|  | Most people at my workplace could take time out of the work day to participate in a group-based program | 0.448 |  |  |  |
| Scale reliability |  |  |  |  | <0.700 |
