# Supplementary material for: Evaluation of Get Healthy at Work, a state-wide workplace health promotion program in Australia
Source: BMC Public Health. 2019 Feb 13;19:183. doi: 10.1186/s12889-019-6493-y (PMC6373144; doi:10.1186/s12889-019-6493-y)
Supplement: Supplementary file 4 — Table S2. Qualitative research sample characteristics of participating businesses. (DOC 36 kb) [file 12889_2019_6493_MOESM4_ESM.doc]

**Supplement file 4**:

**Table 2: Qualitative sample characteristics of participating businesses**

| **Business** | | **Business size** | | **Mode of BHC** | | **Mode of WHP** | | **Program stage completed** | **Key contact position** | **Focus group characteristics** | **Reason for no focus group participation** |  |
| --- | --- | --- | --- | --- | --- | --- | --- | --- | --- | --- | --- | --- |
|  | 1 | | small | | service provider | | online | monitor/review | business owner | office administrative staff: female (n=4) | n/a | |
|  | 2 | | small | | online | | service provider | develop WHP | business owner | no focus group | too small, too busy | |
|  | 3 | | medium | | service provider | | service provider | monitor/review | WHS manager | office administrative staff and technicians: male (n=4) female (n=3) | n/a | |
|  | 4 | | small | | service provider | | service provider | work out needs | business manager | no focus group | WHP not implemented | |
|  | 5 | | medium | | service provider | | service provider | BHC | business manager | no focus group | too busy, no capacity | |
|  | 6 | | large | | service provider | | service provider | monitor/review | HR manager | no focus group | business undergoing major change (merger) | |
|  | 7 | | large | | service provider | | service provider | implement WHP | WHS manager | no focus group | too busy, no capacity | |
|  | 8 | | medium | | online | | service provider | monitor/review | WHS manager |  | n/a | |
|  | 9 | | medium | | online | | service provider | prioritise needs | WHS manager + Team coordinator | office administrative and professional staff: male (n=1) female (n=3) | n/a | |
|  | 10 | | small | | service provider | | service provider | prioritise needs | business manager | no focus group | too small, staff work different days | |
|  | 11 | | Medium | | service provider | | online | BHC | general manager | no focus group | WHP not implemented | |

Legend: BHC= Brief health check. WHP= Workplace health program. WHS= Work Health and Safety. Business 9 included 2 interviewees
