# Supplementary material for: Evaluation of Get Healthy at Work, a state-wide workplace health promotion program in Australia
Source: BMC Public Health. 2019 Feb 13;19:183. doi: 10.1186/s12889-019-6493-y (PMC6373144; doi:10.1186/s12889-019-6493-y)
Supplement: Supplementary file 5 — Table S3. Detailed qualitative data findings. (DOC 53 kb) [file 12889_2019_6493_MOESM5_ESM.doc]

**Supplementary file 5**:

**Table 3- Qualitative results in detail - factors contributing to implementation of Get Healthy at Work**

| **Construct** | **Qualitative finding** | **Quote** |
| --- | --- | --- |
| **Business level factors** | | |
| Motivation | Businesses often encountered GHaW while searching for ways to improve employee health, evidence they were already interested in implementing a WHP. Smaller businesses viewed the company as "family" and their readiness to change the workplace stemmed from their own perspectives of health and perception that their employees’ health was suboptimal. Larger businesses described their reasons for registering for GHaW as an opportunity to gain new ideas to embed into existing WHPS. | *I took it to the staff meeting and said, 'It's not a big deal, but something we can all participate in and something we're all conscious of, and this might sort of motivate us a bit, too, to come on board and see what's involved* (KC, small).  *We noticed the guys buy a lot of chips and pies and things like that at lunch time. So what we were thinking of doing was trying to put a healthy eating programme in place* (KC, medium). |
| Previous WHP experience | Businesses’ experiences with or awareness of WHPs was a large factor in how they engaged with GHaW. Smaller size businesses may have felt ready to implement a WHP but were less familiar with the process or what GHaW involved. They were more in need of guidance and assistance. Larger businesses were more familiar with WHPs, having implemented WHPs previously, and therefore were more likely to cherry pick program elements. | *It was all new to me and I really didn't understand what the process was* (KC, small)  *We have regular health promotion activities on site. … What I'm talking about now is probably more our approach to health promotion as well as Get Healthy at Work* (KC, large). |
| Supportive leadership | Leadership involvement was an important driver for implementation. Leadership actions, such as allowing WHP participation during work hours, and health beliefs, such as feeling a business has a role in employee health, had potential to drive the program. Two FGs spoke of the difficulty getting things moving because of a lack of champion or push from senior level. | *The manager at the time of this was, he was focused on empowering individuals, so we'll get the check done and they will learn something about their health that could assist that* (KC, medium). |
| Business priorities | Organisational priorities and WHP implementation were not always aligned. SPs perceived smaller businesses were unlikely to reach the implementation stage because employee wellbeing was not a high priority. Some KCs stated competing priorities as a reason for their slow progress through the program cycle. FG employees similarly felt business priorities hindered their participation. | *It's probably not as top priority as for me as so many other things I've got to do at the moment, so that's another issue I think* (KC, small). |
| Organisational structures | Corporate structures and changes in the organisation or staffing affected implementation. For businesses with national or international management, WHP implementation at a local level could require a lengthy process of approvals and depend upon what is already in place nationally/ internationally. Participants in one FG spoke of how their involvement with GHaW within their immediate business had flow-on effects (such as a greater interest in healthy eating) to colleagues from other businesses they worked with. | *Every stage they [KC] have to go through a level of approval before they’ll do it and it seems to be a long drawn out process, so no. We haven't noticed that many get through* [the BHC] (SP). |
| **Program characteristics** | | |
| Government delivery | As a government program, GHaW was considered trustworthy, particularly among businesses receiving government funding and/or familiar with other government-led interventions. Employees were favourable toward government involvement in workplace health, explaining it made sense given the amount of time people spend in the workplace and government’s position of influence over businesses’ approaches to employees’ health. Favourability was tempered by concerns about the government's use of for-profit service providers, principally due to poor understanding of the role of SPs and their for-profit approach.  The main reason SPs stated they tendered for GHaW service was because it was a government program. SPs were sentimental about the goodwill they acquire from working with such a program and some mentioned it having a positive effect on staff morale. | *You're always a bit dubious about any of that sort of stuff, but then it was government endorsed and so forth so that obviously helped* (KC, medium).  *Because people spend so much time at their place in employment that the government looked into that back end of that is a good idea* (KC, small).  *It's good to be seen as a company … as this organisation to be aligned with the government and supporting peoples’ health* (SP). |
| Financial incentive | For small businesses, cost was a predominant concern. The financial incentive offered by GHaW to subsidise WHP equipment had a positive effect on enabling and encouraging businesses to participate. Perspectives on the cost of running the program changed from being a barrier to an expense justified by the observed benefits, but for businesses unable to achieve the participation requirements in order to receive the incentive, cost became a disincentive. | *This is something we've had discussions about in different groups is small businesses ... struggling to survive most the time let alone thinking about putting more money into programmes that ... to support their workers when they're often feeling marginalised as an employer anyway* (KC, small). |
| Health priority focus | The GHaW approach is to focus on a specific health priority (e.g., diet or physical activity but not both) and to treat tobacco control as the top priority, yet businesses wanted and implemented WHPs that encompassed health more widely and was inclusive of the needs of all employees. This presented a challenge for SPs caught between the businesses’ wants and GHaW directive. | *It's been difficult getting companies to get on board to do smoking cessation programmes. …they see it as, I want to be offering something to the majority of my staff. ... I want to run a nutrition workshop. I want to get a better attendance, there'll be more people engaged with it* (SP).  *Everyone's done an online health check, they've come up with an individual risk factor, and yet we're not necessarily targeting that because we have to do something for everybody* (KC, small). |
| A sequential program cycle | The program cycle was either not known or poorly understood; many KCs perceived the BHC was the GHaW program or were unaware of steps beyond the BHC. Completion of the BHC was essential for moving on to the next stage however for many businesses, especially for multi-site business, the logistics of organising employees to complete the BHCs was a challenge. While the online BHC option provided more flexibility, staff computer access and literacy skills could be challenges. | *We planned to get to 50 so that we would get the report, so then we could use that information to maybe tailor a programme if there was a need for it. But because we didn't get to 50, we didn't get the report ... there was no tailored programme* (KC, medium). |
| Sustainable action plans | While the program encourages a mix of people, place and policy initiatives, activity-based interventions (such as a weekly exercise activity, gym equipment) appeared to attract small levels of participation. Policies (such as changes from unhealthy to healthy food catering options implemented by many of the groups) appeared to be more encompassing. The reason for each element was not always understood by KCs or possibly not well communicated. | [Participation in exercise activity is] *a bit over half. Yeah. Like everything, a few people drop off, and get lazy. Three months is everyone goes really hard for the first few months, and it starts to drop off* (FG, medium). |
| **Program delivery** | | |
| Service provider support | The majority of businesses spoke of a long time lag before they were contacted by a SP, resulting in waning momentum and enthusiasm. Yet businesses that did receive the contact they desired spoke positively about the SP and program. Small businesses appeared to have a greater need for program implementation support but were unsure of the SP’s role in the GHaW program. | *I was still very open to it, but I think I was a bit more like, "There's no urgency from that side, so there's no urgency from my side,"…I think after I signed up for it, …somebody rang me …we've talked about, like why I signed up… I didn't hear anything more about that* (KC, small). |
| Information technologies and processes | SPs mentioned how initial problems in accessing the GHaW portal system impeded their ability to contact new clients at registration and sometimes assigned a business recruited by one SP to their competitor. SPs also experienced problems submitting invoices. Early processes for uploading BHCs were manual and went through third parties. Changes were made to automate the process during the evaluation period.  SPs all mentioned the challenge of developing WHPs for businesses without access to the business’s health survey results which might illuminate the key health issues for the workplace and allow them to better tailor action plans, or even to know to start the next stage of the program cycle. Some KCs also commented that the action plans were too generic to meet their individual needs. | *This would be easy now that they've gone online. But previously the sending of the questionnaires to a certain document management group who would scan the things and put it up online into the profile was tedious and made things difficult* (SP).  *We try to give them all of those examples that can happen, but I think also the problem is we don’t know what we are going to be able to give them at the end of it for the action plan* (SP).  *And a lot of the businesses can't find it. So they go, "Nah I didn't get it". But it probably is sitting there on the website; they just don't know where to find it* (SP). |
| Communication | Businesses found program information provided on the GHAW web portal or through SPs unclear and confusing; small businesses in particular were uncertain about program steps and content. SPs believed their earlier involvement in the program cycle and clearer website information would help reduce some of the confusion.  SPs also reported difficulties when escalating issues. This may result from undefined or ineffective communication channels as at times SPs mention escalating issues to two separate State government departments involved at the higher level of implementation. | *I think from those smaller businesses that are really wanting the programme… they've just been overwhelmed, just from the get-go... If the initial contact with them, with the Get Healthy site wasn't so overwhelming, we may have a better chance of going forward with them* (SP).  *It was never very clear about how the system works, I really didn't understand how [SP] plugged into Get Healthy at Work. Those sort of things that you had these separate providers and it wasn't terribly clear* (KC, small). |
| Service capacity | Some SPs mentioned logistical issues of agreeing to contact one business located in a certain part of the state, only to find that the business was primarily based in another location that might be harder to reach. Many SPs felt that the program ran a high operational cost due to the time and resources they needed to commit in order to bring a business to a stage where the SP could offer their for-profit services. Further, the financial incentives offered by GHaW to workplaces to support the WHP are unlikely to cover many of the SPs’ for-profit services. | *We're just not even getting to the point where we're delivering an action plan so we can offer those services. Because we're not actually allowed to offer them until the action plan’s completed* (SP). |

*Legend: BHC – Brief Health Check , GHaW – Get Healthy at Work, FG – Focus groups, KC – key business contact, SP – Service Providers*
